# Metabolic profiles of saliva in male mouse models of chronic sleep disorders induced by psychophysiological stress

**DOI:** 10.1038/s41598-023-38289-1

**Published:** 2023-07-10

**Authors:** Katsutaka Oishi, Yuhei Yajima, Yuta Yoshida, Hideo Hagihara, Tsuyoshi Miyakawa, Sayaka Higo-Yamamoto, Atsushi Toyoda

**Affiliations:** 1 Healthy Food Science Research Group, Cellular and Molecular Biotechnology Research Institute, National Institute of Advanced Industrial Science and Technology (AIST), Central 6, 1-1-1 Higashi, Tsukuba, Ibaraki 305-8566 Japan; 2 Department of Applied Biological Science, Graduate School of Science and Technology, Tokyo University of Science, Noda, Chiba Japan; 3 Department of Computational Biology and Medical Sciences, Graduate School of Frontier Sciences, The University of Tokyo, Kashiwa, Chiba Japan; 4 School of Integrative and Global Majors (SIGMA), University of Tsukuba, Tsukuba, Ibaraki Japan; 5 College of Agriculture, Ibaraki University, Ami, Ibaraki Japan; 6 Ibaraki Prefecture Livestock Research Center, Ishioka, Ibaraki Japan; 7 United Graduate School of Agricultural Science, Tokyo University of Agriculture and Technology, Fuchu, Tokyo Japan; 8 Division of Systems Medical Science, Center for Medical Science, Fujita Health University, Toyoake, Aichi Japan

**Keywords:** Homeostasis, Neurophysiology

## Abstract

Disordered sleep is a global social problem and an established significant risk factor for psychological and metabolic diseases. We profiled non-targeted metabolites in saliva from mouse models of chronic sleep disorder (CSD). We identified 288 and 55 metabolites using CE-FTMS and LC-TOFMS, respectively, among which concentrations of 58 (CE-FTMS) and three (LC-TOFMS) were significantly changed by CSD. Pathway analysis revealed that CSD significantly suppressed glycine, serine and threonine metabolism. Arginine and proline metabolic pathways were among those that were both upregulated and downregulated. Pathways of alanine, aspartate and glutamate metabolism, genetic information processing, and the TCA cycle tended to be downregulated, whereas histidine metabolism tended to be upregulated in mice with CSD. Pyruvate, lactate, malate, succinate and the glycemic amino acids alanine, glycine, methionine, proline, and threonine were significantly decreased, whereas 3-hydroxybutyric and 2-hydroxybutyric acids associated with ketosis were significantly increased, suggesting abnormal glucose metabolism in mice with CSD. Increases in the metabolites histamine and kynurenic acid that are associated with the central nervous system- and decreased glycine, might be associated with sleep dysregulation and impaired cognitive dysfunction in mice with CSD. Our findings suggested that profiling salivary metabolites could be a useful strategy for diagnosing CSD.

## Introduction

Recognition of the importance of sleep as a social, as well as a health problem has increased interest in sleep and sleep research worldwide. Chronic sleep disorders (CSDs) are associated with significant and cumulative neurobehavioral deficits and physiological changes that result in hypertension, obesity, diabetes, cardiovascular morbidity, and stroke in addition to psychiatric disorders such as depression^1, 2^. Sleep disorders confer a major burden on society due to their high prevalence and association with socio-economic losses due to absenteeism and increased rates of workplace accidents^3, 4^. Sleep problems in the clinical setting have generally been evaluated using subjective questionnaires such as the Pittsburgh Sleep Quality Index (PSQI)^5^ and the St. Mary's Hospital Sleep Questionnaire^6^. However, putative biomarkers of sleep problems have been revealed in blood, urine and feces by approaches such as transcriptomics, miRNA omics, proteomics, and metabolomics^7–9^.

Saliva is a non-invasive, cost-efficient way to monitor health. It is easily sampled and does not require trained professionals. Saliva contains metabolites produced by the salivary gland, and provides a significant amount of information^10, 11^. Changes in the brain are more precisely reflected by levels of numerous substances in saliva than in blood^12, 13^. Salivary components can be biomarkers of pathological states such periodontal disease, oral, breast and pancreatic cancers^14^, and mental disorders^15^, as well as Parkinson^16^ and Alzheimer^17^ diseases. Salivary concentrations of amyloid-β^14, 18^ and tau^19^ reflect cognitive dysfunction. Proteomic profiling has revealed that neurodegenerative and inflammatory pathways are enriched in tongue tissues from rats with chronic sleep deprivation^20^. However, the effects of CSD on salivary metabolic profiles remain unknown.

Sleep, along with its disorders and other problems have been extensively studied in humans but are rather complex by nature. Therefore, studies of other animals have been invaluable in terms of increasing understanding about sleep physiology and the underlying mechanisms of sleep disorders^21^. We developed models of CSD by causing mice to perpetually avoid water by remaining on running-wheels that result in psychophysiological stress^22–26^. This is an important risk factor that contributes to the mechanisms of underlying sleep disorders in humans^27^. Mouse models of CSD are characterized by reduced amplitude of circadian rhythms such as wheel-running activity and sleep–wake cycles, sleep fragmentation, and hyperphagia for several weeks without adaptation^22–26^. Plasma levels of catecholamines (adrenaline and noradrenaline) are significantly elevated, whereas those of corticosterone are only slightly affected in mice with CSD, suggesting activation of the sympathetic-adrenal medullary axis^23^. We previously showed that CSD impairs recognition memory and elicits anxiety-like behavior during novel object recognition and open field tests, respectively^26^. Mice with CSD notably develop hyperphasia accompanied by hypoleptinemia and impaired glucose tolerance^24, 25^, which resembles the situation in insomniac humans^28^. These findings suggested that our CSD model mice would be useful for studying the mechanisms underlying neurobehavioral difficulties and metabolic disorders caused by sleep disorders. Here, we profiled the salivary metabolomes of control and CSD mice to identify metabolites associated with CSD-induced psychological and metabolic disorders.

## Results

### Circadian wheel-running activity and sleep–wake rhythms

**Figure 1:**
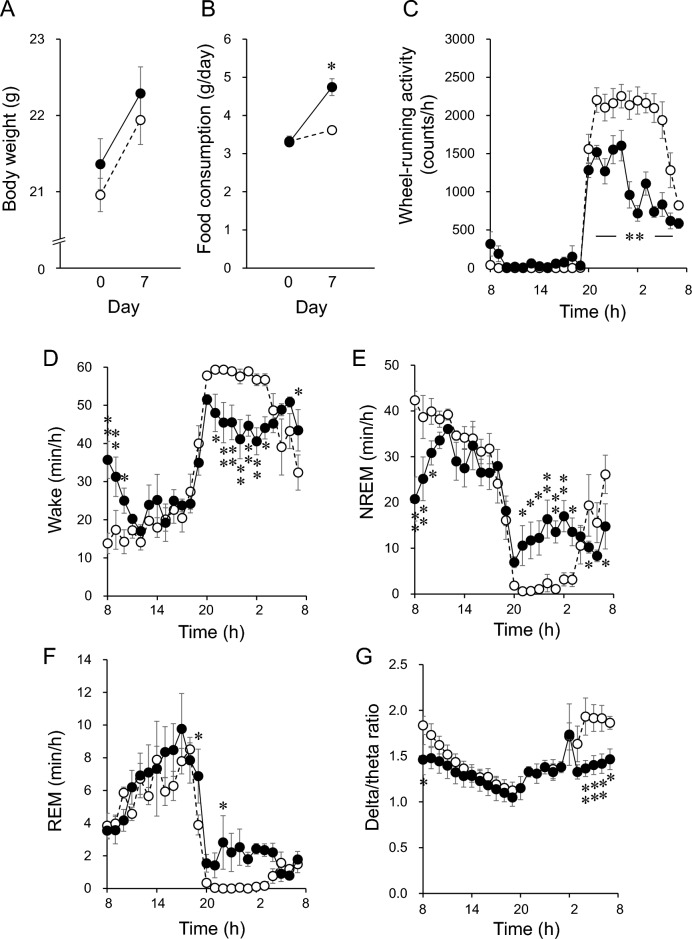
Effects of CSD on body weight gain, food consumption, circadian wheel-running and sleep–wake rhythms. (**A**) Body weight gain (n = 6). (**B**) Daily food consumption (n = 6). (**C**) Circadian rhythm of wheel-running activity (control, n = 6; CSD, n = 4). (**D–F**) Circadian rhythm of wakefulness (**D**), NREM (**E**) and REM (**F**) sleep duration (n = 4). (**G**) Circadian EEG delta/theta ratios during NREM sleep in mice (n = 4). Wheel-running activity (**C**) and sleep (**D–G**) data were averaged for last 72 h of experimentation. Data are shown as means ± SEM. Unfilled and filled circles indicate values for control and CSD mice, respectively. Significant differences between control and CSD mice at each time point. Horizontal unfilled and filled bars indicate light and dark periods, respectively. *P < 0.05, **P < 0.01.

Daily food intake was increased by 31% at seven days after inducing CSD, although gains in body weight did not significantly differ between CSD and control mice (Fig. 1A, B). Nighttime and daytime activities of CSD mice respectively decreased 0.56-fold and increased 21.8-fold (Fig. 1C). Disrupted circadian wheel-running activity by the CSD mice persisted without adaptation and exhaustion throughout the study.

Circadian sleep–wake rhythms were precise in control mice, with the nocturnal mice sleeping more during the light, than the dark period. Sleep–wake rhythms (Fig. 1D‒F) and circadian wheel-running activity were similarly damped in CSD mice. Seven days of CSD increased wakefulness and decreased NREM sleep during the start of the light period when mice usually fall asleep (Fig. 1E). In contrast, the duration of non-rapid eye movement (NREM) sleep and wakefulness respectively increased and decreased during the second half of the dark period. These findings corresponded to the mice having difficulty falling asleep during the light period and being sleepy during the active phase, which is typical of the insomnia phenotype. The duration of rapid eye movement (REM) sleep increased during the active phase (Fig. 1F). Figure 1G shows a significant decrease in the EEG delta:theta ratio in NREM sleep during the last half of the dark period under CSD.

### Salivary metabolic profiles in CSD mice

**Figure 2:**
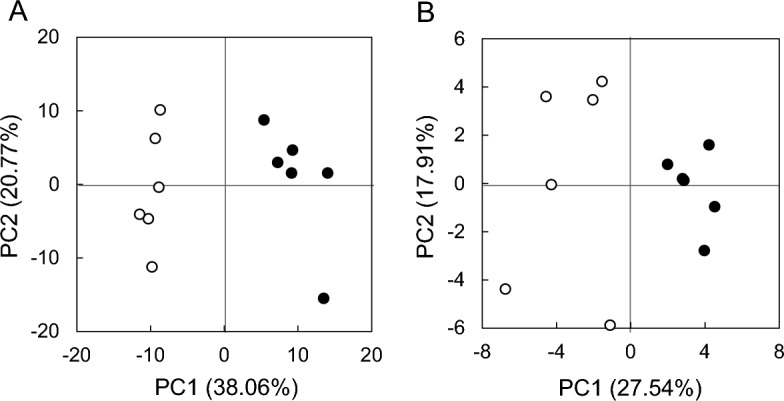
Principal component analysis (PCA) scores. Principle components 1 and 2 in saliva of control and CSD groups analyzed by CE-FTMS (**A**) and LC-TOFMS (**B**). Unfilled and filled circles indicate control and CSD mice, respectively.

We identified 288 and 55 metabolites in the salivary metabolomes from control and CSD mice using capillary electrophoresis-Fourier transform mass spectrometry (CE-FTMS) and liquid chromatography time-of-flight mass spectrometry (LC-TOFMS) in cation and anion modes and LC-TOFMS in positive and negative modes, respectively. Among them, the concentrations of 58 (*p* < 0.05 and *q* < 0.05, CE-FTMS) (Table 1 and Supplemental Tables 1 and 2) and three (*p* < 0.05 and *q* < 0.05, LC-TOFMS) (Table 2 and Supplemental Table 3) were significantly affected by CSD. Several other metabolites determined by CE-FTMS were unique to CSD (21 species, including acetoacetic acid, kynurenic acid, biopterin, and 4-pyridoxic acid) (Table 3) and control (17 species, including sarcosine, decanoic acid, fumaric acid, and glyoxylic acid) mice (Table 4). Principal component analysis of the metabolome profiles generated by CE-FTMS and by LC-TOFMS revealed a clear separation between the CSD and control mice (Fig. 2). Plotted PCA scores (Fig. 2A) revealed negative and positive PC1 scores for the control and CSD mice, respectively. This suggested that the PC1 score positively correlated with the CSD effect. The factor loading values of 65 and 11 of all metabolites detected by CE-FTMS were respectively positive (*r* > 0.8) and negative (*r* < − 0.8), indicating a close correlation with PC1(Supplemental Table 4).

**Table 1:** Significantly altered salivary metabolites in CSD mice profiled by CE-FTMS.

Metabolite	Fold change^a^	*p*	*q*
11-Aminoundecanoic acid	10.1	< 0.0001	< 0.0001
Lactic acid	0.6	< 0.0001	< 0.0001
Urea	1.6	< 0.0001	0.0004
3-Indoxylsulfuric acid	9.0	< 0.0001	0.0008
S-Adenosylmethionine	2.3	< 0.0001	0.0008
Homovanillic acid 3,4-Dihydroxyhydrocinnamic acid Hydroxyphenyllactic acid	4.3	< 0.0001	0.0015
3-Hydroxybutyric acid	4.1	0.0001	0.0031
4-Aminohippuric acid	7.6	0.0002	0.0035
*N*-Formylmethionine	3.9	0.0001	0.0035
*N*1-Methyl-4-pyridone-5-carboxamide	2.3	0.0002	0.0035
2-Hydroxybutyric acid	2.8	0.0002	0.0037
Succinic acid	0.5	0.0002	0.0038
Malic acid	0.7	0.0003	0.0044
Phenaceturic acid	6.8	0.0003	0.0052
Allantoin	2.7	0.0004	0.0052
*N*-Acetylhistidine	4.0	0.0004	0.0054
7-Methylguanine	3.3	0.0005	0.0058
Gluconic acid	2.5	0.0006	0.0061
*N*1-Methylguanosine	3.0	0.0005	0.0061
Creatinine	2.7	0.0006	0.0064
5-Hydroxypentanoic acid	2.0	0.0008	0.0076
Uridine	2.6	0.0009	0.0082
Guanidoacetic acid	3.1	0.0011	0.0097
Putrescine	2.0	0.0011	0.0097
Thr	0.5	0.0012	0.0098
Imidazole-4-acetic acid	3.3	0.0014	0.0112
*N*-Acetylleucine-1 Isovalerylalanine-1	5.0	0.0017	0.0125
Methionine sulfoxide	0.6	0.0017	0.0126
1-Methyl-4-imidazoleacetic acid	8.7	0.0019	0.0130
6-Aminohexanoic acid	1.7	0.0019	0.0130
Isethionic acid	2.4	0.0022	0.0143
Gly-Asp	1.5	0.0023	0.0144
*N*-Acetylglycine	2.1	0.0023	0.0144
Ser-Glu	2.1	0.0024	0.0144
1-Methylhydantoin Glycine anhydride	2.3	0.0026	0.0145
Choline	0.5	0.0026	0.0145
Histamine	3.0	0.0026	0.0145
1-Methyladenosine	2.2	0.0029	0.0150
Hydroxyproline	0.4	0.0029	0.0150
4-(β-Acetylaminoethyl)imidazole	9.8	0.0033	0.0169
Ala	0.6	0.0037	0.0181
Pipecolic acid	1.9	0.0038	0.0181
XA0012	3.2	0.0038	0.0181
Mucic acid	0.3	0.0040	0.0188
*N*-Acetylputrescine	2.4	0.0042	0.0189
Gluconolactone	2.3	0.0048	0.0215
Glutaric acid Methylsuccinic acid	0.6	0.0051	0.0222
SDMA	2.0	0.0055	0.0232
XC0089 Nicotinamide riboside	1.4	0.0056	0.0232
Muscimol	1.9	0.006	0.0232
XA0035	3.7	0.006	0.0233
Pro	0.5	0.006	0.0240
Cysteic acid	0.5	0.006	0.0246
Cadaverine	5.6	0.007	0.0256
*N*8-Acetylspermidine	1.8	0.007	0.0258
Cysteinesulfinic acid	0.6	0.008	0.0300
Ascorbic acid	9.1	0.009	0.0307
Thymine	2.1	0.009	0.0307
Glucuronic acid-1 Galacturonic acid-1	3.2	0.009	0.0321
2-Hydroxyoctanoic acid 8-Hydroxyoctanoic acid	2.2	0.009	0.0324
4-Guanidinobutyric acid	2.7	0.010	0.0324
γ-Glu-Lys_divalent	0.5	0.010	0.0324
1-Methylnicotinamide	2.1	0.011	0.0338
1H-Imidazole-4-propionic acid	2.4	0.011	0.0338
ADMA	1.6	0.011	0.0338
Imidazolelactic acid	1.7	0.011	0.0338
Pyruvic acid	0.7	0.011	0.0338
Met	0.5	0.012	0.0361
Decarboxylated *S*-adenosylmethionine	2.3	0.012	0.0372
Gly	0.7	0.015	0.0433
1-Methylhistamine	1.9	0.017	0.0485
Carboxymethyllysine	1.8	0.017	0.0494
Glycerophosphocholine	0.5	0.017	0.0494

^a^CSD vs. control.

**Table 2:** Significantly altered salivary metabolites in CSD mice profiled by LC-TOFMS.

Metabolite	Fold change^a^	*p*	*q*
Kynurenic acid	2.9	0.0001	0.0052
AC (18:1)	2.2	0.0020	0.0305
Riboflavin	0.7	0.0022	0.0305

^a^CSD vs. control.

**Table 3:** Unique salivary metabolites in CSD mice profiled by CE-FTMS.

Metabolite	
4-Hydroxy-3-methoxyphenylglycol sulfate	
4-Pyridoxic acid	
5-Methoxyindoleacetic acid-1/indole-3-lactic acid-1	
7,8-Dihydrobiopterin	
Acetoacetic acid	
Biopterin	
cIMP	
Guanidinosuccinic acid	
Hippuric acid	
Kynurenic acid	
Malonylcarnitine	
Methylguanidine	
*N*-Acetylglutamine	
*N*-Acetyltyrosine	
*N*-Glycolylneuraminic acid	
Nalidixic acid	
*p*-Hydroxymandelic acid/Homogentisic acid	
Sulfotyrosine	
Tyr-Glu	
XA0017	
XC0065	

Detected in ≥ 50% of CSD mice, but not in any controls.

**Table 4:** Unique salivary metabolites in control mice profiled by CE-FTMS.

Metabolite	
2,4-Diaminobutyric acid	
2-Oxoadipic acid	
3,4-Dihydroxyphenylglycol	
4-Hydroxyphenylacetaldehyde	
Azetidine 2-carboxylic acid	
Decanoic acid	
Fumaric acid	
Glyoxylic acid	
Isovalerylcarnitine	
*N*-Acetylglucosylamine	
*N*-Formylaspartic acid	
*N*-Formylglycine	
Phytic acid_divalent	
Pyridoxine	
Sarcosine	
γ-Glu-Asn	
γ-Glu-Thr	

Detected in ≥ 50% of control, but not in any CSD mice.

We implemented statistical hypothesis tests of factor loading in PC1 to determine the effects of CSD on metabolic pathways in mouse saliva and identified 147 statistically significant metabolites (*q* < 0.05). We applied an independent Metabolite Set Enrichment Analysis (MESA) of significantly positive and negative metabolites in PC1. The results showed that CSD significantly suppressed the glycine, serine and threonine metabolism pathway (Table 5). The arginine and proline metabolism pathway was among upregulated and downregulated pathways. Pathways of alanine, aspartate and glutamate metabolism, genetic information processing, and TCA cycle tended to be downregulated, whereas histidine metabolism tended to be upregulated by CSD.

**Table 5:** Results of metabolic pathway analysis.

Metabolic pathway	Total^a^	Hits^b^	*p*^c^	*p*^d^
Upregulated				
Histidine	16	4	0.00201	0.169
Arginine and proline	38	5	0.0103	0.435
Downregulated				
Glycine, serine and threonine	33	5	0.000071	0.00596
Arginine and proline	38	4	0.00175	0.0736
Alanine, aspartate and glutamate	28	3	0.0068	0.19
Aminoacyl-tRNA biosynthesis	48	3	0.0298	0.544
Citrate cycle (TCA cycle)	20	2	0.0324	0.544

^a^Total number of compounds in pathway.

^b^Number (n) that matched uploaded user data.

^c^Original *p* calculated from enrichment analysis.

^d^Adjusted *p* using false discovery rate.

Pyruvate, lactate, malate, succinate and the glycemic amino acids alanine, glycine, methionine, proline, and threonine were significantly decreased, whereas the ketosis-associated compounds 3-hydroxybutyric acid, 2-hydroxybutyric acid, 4-methyl-2-oxovaleric acid and 3-methyl-2-oxovaleric acid were significantly increased by CSD (Table 1). *S*-adenosylmethionine (SAM) and its decarboxylated (dc) SAM) concentrations were both increased 2.3-fold in CSD mice, which would account for increases in the methylated metabolites, 1-methyl-4-imidazoleacetic acid, 1-methyladenosine, 1-methylhydantoin, 7-methylguanine, ADMA and SDMA, and polyamines such as putrescine, *N*-acetylputrescine, cadaverine, *N*-methylputrescine and *N*^8^-acetylspermidine. In addition to these polyamines, the central nervous system (CNS)-associated metabolites, histamine, homovanillic acid and kynurenic acid were robustly increased in CSD mice (Tables 1 and 2). Sarcosine is an *N*-methyl-*D*-aspartate (NMDA) receptor co-agonist that was reduced to undetectable levels in CSD mice, whereas the concentration in control mice was 1.2 ± 0.18 μM (Supplemental Table 2). The guanidino compounds guanidoacetic and 4-guanidinobutyric acids, which induce seizures and convulsions in experimental animals, were 3.1- and 2.7-fold increased, respectively, in CSD mice (Table 1).

### Systemic inflammation in CSD mice

Chronic sleep deprivation induces oral and systemic inflammation in rats^20^. Therefore, we assessed IL-6 and IL-1β mRNA expression in control and CSD tongue tissues and antigen levels in plasma. We found that they were identical between the tongue tissues (data not shown), although plasma IL-6 concentrations were significantly higher in CSD, than control mice (25.2 ± 4.9 *vs.* 7.7 ± 1.4 pg/mL; *P* < 0.01).

### Lactate levels in saliva, plasma and whole brain

**Figure 3:**
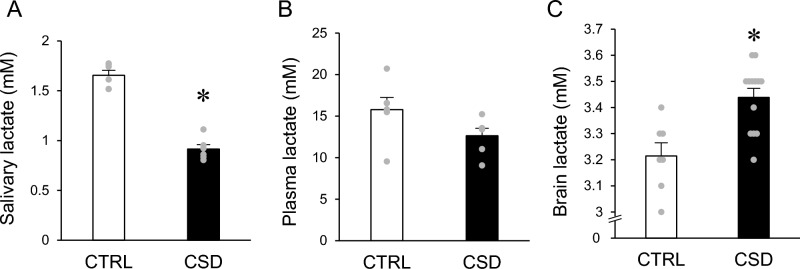
Saliva, plasma, and brain lactate concentrations. (**A–C**) Saliva, plasma, and brain, respectively. Data are shown as means ± SEM (saliva: control, n = 7; CSD, n = 13; plasma: control, n = 6; CSD, n = 6; brain: control, n = 7; CSD, n = 13. Control and CSD mice significantly differed. *P < 0.01.

Lactate might a biomarker for sleep–wake status, because increased cortical lactate concentrations closely correlate with the waking state in mice^29^. Our results showed that lactate was respectively decreased in saliva and significantly increased in the brains of CSD model mice (Fig. 3A,C) and similar in plasma between control and CSD mice (Fig. 3B). Levels of lactate were much lower in saliva than in blood.

## Glycine and sarcosine levels in saliva, plasma, and whole brains of mice

**Figure 4:**
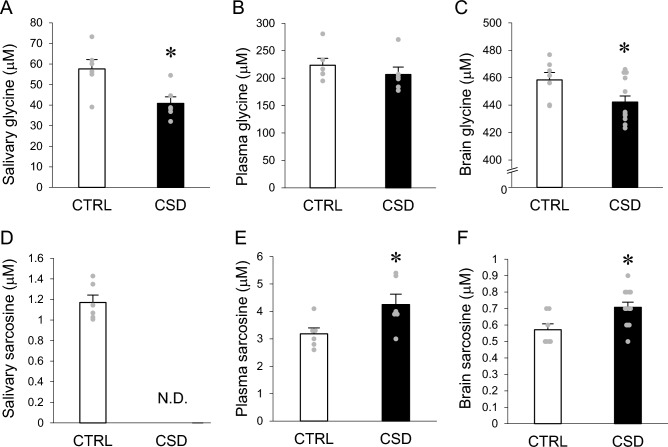
Glycine and sarcosine concentrations in saliva, plasma, and brain. (**A–C**) Glycine concentrations in saliva, plasma, and brain, respectively. (**D–F**) Sarcosine concentrations in saliva, plasma, and brain, respectively. Data are shown as means ± SEM (saliva: control, n = 7; CSD, n = 13; plasma: control, n = 6; CSD, n = 6; brain: control, n = 7; CSD, n = 13. Control and CSD mice significantly differed. *P < 0.05.

Glycine and sarcosine were involved in the modulation of NMDA receptor activity. Therefore, we assessed levels of these amino acids in whole brains, plasma and saliva of CSD and control mice. The CSD significantly decreased glycine concentrations in the brains and saliva, but not significantly in plasma (Fig. 4A‒C). However, glycine concentrations were ~ 10-fold higher in brains than saliva. Sarcosine concentrations were significantly increased by the CSD in brains and plasma, but were reduced to undetectable levels in saliva (Fig. 4D‒F).

## Discussion

We applied non-targeted metabolic profiling of saliva from mouse models of CSD^22–26^ using CE-FTMS and LC-TOFMS and identified 288 and 55 metabolites, respectively. Among them, the concentrations of 58 (CE-FTMS) and 3 (LC-TOFMS) metabolites were significantly changed by CSD. Pathway analysis revealed that CSD significantly suppressed glycine, serine and threonine metabolism. The arginine and proline metabolism pathway was both upregulated and downregulated, whereas alanine, aspartate and glutamate metabolism, genetic information processing, and TCA cycle pathways tended to be downregulated, and histidine metabolism tended to be upregulated by CSD. The downregulated TCA cycle and glycemic amino acid pathways suggested impaired glucose metabolism in CSD mice. Increased polyamines and the CNS-related metabolites, histamine, homovanillic acid and kynurenic acid might be associated with sleep dysregulation and impaired cognitive dysfunction in CSD mice. Our findings suggested that profiling salivary water-soluble metabolites could be useful for diagnosing CSD.

Pyruvate, lactate, malate, succinate and the glycemic amino acids alanine, glycine, methionine, proline, and threonine were significantly decreased by CSD, whereas ketosis-related 3-hydroxybutyric, 2-hydroxybutyric, 4-methyl-2-oxovaleric and 3-methyl-2-oxovaleric acids were significantly increased, again suggesting that glucose metabolism is abnormal in CSD mice. We previously revealed significantly decreased plasma levels of alanine, glycine, methionine, and threonine in CSD mice during the sleep phase^24^. The present results suggested correlations between the effects of CSD on salivary and plasma amino acid concentrations. Glycine and sarcosine are involved in modulating NMDA receptor activity and were significantly reduced by the CSD in saliva. Here, we showed that glycine levels were also significantly reduced in whole brains of CSD mice. In contrast, sarcosine concentrations were significantly increased by CSD in the brain, but reduced to undetectable levels in saliva. Glycine is involved in sleep regulation via peripheral vasodilation by activating NMDA receptors in the suprachiasmatic nucleus (SCN) in the central circadian clock in mammals^30^. Injected glycine improves sleep quality by decreasing core body temperatures in humans^31^. Since the concentration of glycine in the brain was over two orders of magnitude higher than that of sarcosine, the decreased glycine concentration apparently has a substantial physiological effect on the brain. The present findings suggested that decreased concentrations of glycine in cerebrospinal fluid are at least partly involved in the sleep phenotype in CSD mice. Changes in salivary glycine levels might reflect those in the brain, although glycine concentrations might be affected only in specific brain regions.

Concentrations of SAM and its decarboxylated (dc) form were increased 2.3-fold in CSD mice, which would account for increased levels of the methylated metabolites, 1-methyl-4- imidazoleacetic acid, 1-methyladenosine, 1-methylhydantoin, 7-methylguanine, symmetric dimethylarginine (ADMA) and symmetric dimethylarginine (SDMA), as well as the polyamines putrescine, *N*-acetylputrescine, cadaverine, *N*-methylputrescine and *N*^8^-acetylspermidine. Salivary concentrations of spermidine, which protects against neurodegeneration^32^, were significantly decreased in CSD mice, regardless of the significant increase in putrescine. Polyamine metabolism, such as the conversion of putrescine by spermidine synthase into spermidine might be disrupted in CSD mice. Excess SAM disturbs circadian rhythms of behavior and clock gene expression by inhibiting normal methylation via its catabolism to adenine and methylthioadenosine in mice^33^. The accumulation of SAM might be involved in phenotypes such as disrupted circadian sleep–wake cycles, anxiety-like behavior and impaired long-term memory in CSD mice.

In addition to polyamines, histamine, homovanillic acid and kynurenic acid that are associated with the CNS were robustly increased in CSD mice. Histamine plays a pivotal role in sleep–wake regulation via H1 and H3 receptors^34^. Histamine or H1 receptor agonists induce wakefulness, whereas the H3 receptor functions as an auto-receptor and regulates histamine synthesis and release. Histamine release in the hypothalamus and other target regions is maximal during wakefulness. Increased salivary histamines in CSD mice might reflect central histamine dysregulation, although evidence has not yet linked salivary histamine with sleep. Concentrations of the dopamine metabolite, homovanillic acid, are elevated in the cerebrospinal fluid of patients with depression and comorbid posttraumatic stress disorder^35^. However, lower concentrations of homovanillic acid in cerebrospinal fluid might serve as a biomarker of depression^36, 37^. Intermittent tail-shock stress increases extracellular dopamine relative to the baseline in the striatum, nucleus accumbens, and medial frontal cortex accompanied by increased homovanillic acid concentrations in rats; this suggested that stress increases dopamine release and metabolism^38^. Elevated salivary homovanillic acid suggests global activation of the dopaminergic system in CSD mice. Kynurenic acid, an endogenous antagonist of α7 nicotinic receptors and ionotropic glutamate receptors are potential salivary biomarkers for patients with schizophrenia and distress intolerance^39, 40^. Our findings suggested that salivary CNS-related metabolites such as histamine, homovanillic acid and kynurenic acid could be biomarkers of CSD caused by psychophysiological stress. Levels of the guanidino compounds guanidoacetic and 4-guanidinobutyric acids that induce seizures and convulsions in experimental animals, were respectively increased 3.1- and 2.7-fold in CSD mice. These findings might also account for the sleep dysregulation, impaired cognitive dysfunction, and increased anxiety-like behavior^26^ in our CSD model mice.

We conducted a pilot study of the effects of restricting sleep from 8 to 5 h for one week on salivary metabolites in healthy persons. The CE-FTMS results revealed only a slight change in salivary metabolites (data not shown). However, we also identified an unusual metabolic profile in a participant with a PSQI of 6, which was considerably higher than of the others (PSQI ≤ 3; data not shown). Furthermore, salivary metabolites except for lactate were only slightly affected in a mouse model of social defeat stress^41^. These results suggested that serious sleep disorders affect salivary metabolic profiles whereas simple sleep restriction or a depression-like state does not.

Extracellular lactate concentrations in the brain comprise an early indicator of wakefulness because arousal rapidly elevates cortical lactate, which is maintained during enforced sleep deprivation^29^. We previously showed that lactate levels in the brain are increased in mouse models of chronic schizophrenia, bipolar, and autism spectrum disorders^42^. Recent large-scale meta-analyses have confirmed increased lactate levels in schizophrenia and bipolar disorder^43, 44^. The present study found significantly decreased salivary, and increased brain lactate concentrations in CSD mice. We collected saliva from the oral cavity of CSD mice at 22:00, when the active phase began. The results of electroencephalography (EEG) revealed decreased and increased brain lactate concentrations while awake and during NREM sleep at 22:00. Therefore, the increase in brain lactate levels in CSD mice seemed to be caused by chronic sleep impairment and not by awakening. Others have found contradictory effects of lactate levels between the brain and saliva in mouse models of social defeat stress^41, 45^; the present findings were similar. Salivary lactate appears to be derived from host circulation via the salivary glands rather than microbes, because lactate concentrations are identical between whole mouth and parotid saliva^46^. The relatively low lactate levels in saliva likely reflect baseline lactate entering the salivary glands from the circulation^46^. Decreased lactate concentrations in saliva might serve as a biomarker for increased levels in the brain, although the underlying mechanisms await discovery.

One limitation of the present study is that the sources of metabolites await identification. The oral microbiota significantly contributes to the salivary metabolome by generating or consuming salivary metabolites^46^. In addition to short-chain fatty acids, concentrations of which are minimal in salivary glands, amines, amino and organic acids reflect microbial metabolic activity^46^. Chronic sleep deprivation induces subtle changes in the oral microbiota of rats and might be responsible for oral inflammation^20^. Salivary concentrations of hydroxyproline derived from collagen breakdown that increase due to neutrophil activation under gingivitis^47^, were significantly decreased under CSD, suggesting suppressed collagen turnover. Changes in the composition of the oral microbiota might have been involved in the effects of CSD on salivary metabolic profiles in the present study, despite the absence of oral inflammation in CSD mice.

This study has some other limitations. We used only male CSD mice, although sex differences in sleep problems have been suggested^48^. Gender differences also affect salivary metabolites including glycine and lactate^10^. Furthermore, the contents of stimulated and unstimulated saliva might differ.

Salivary metabolites upon waking are generally more concentrated than at other times throughout the day^49–51^. Some of this might be due simply to reduced salivary flow at the time of collection compared with other times of the day^49^. However, intra-day salivary metabolomic profiles significantly vary independently of the salivary flow^51^. Notably, ~ 15% of identified salivary metabolites is under circadian control when a routine protocol is constant; this indicates a direct effect of the endogenous circadian clock on salivary metabolic profiles independently of sleep–wake and feeding rhythms^50^. We sampled saliva only at 2 h after activity onset (22:00) to detect the effects of CSD on metabolites, but they appeared to depend on the time of day^51^.

We measured corticosterone levels in saliva since salivary glucocorticoid levels have been widely used as a biological marker of a stress reaction^52^. We found that CSD substantially increased corticosterone levels in saliva and plasma (Supplemental Fig. 1), whereas CSD increased only slightly in plasma in previous studies^23, 25^. However, these studies proceeded under ad libitum feeding, whereas we fasted the CSD mice before sampling. Hypercorticosteronemia might be caused under fasting by hypoleptinemia-induced orexigenic activity in the CSD mice^23–25^. These findings indicate that salivary corticosterone might be inappropriate as a biomarker for sleep disorders in mice under food deprivation condition. The substantial increases of corticosterone in saliva and plasma raise concerns that acute physical stress caused by the food deprivation may affect salivary metabolic profiles and that the results may not solely reflect the effects of CSD.

Saliva is easily accessible and useful for diagnostic purposes. The present results support the notion that salivary metabolites could serve as biomarkers of CSD. Salivary gland-derived brain-derived neurotrophic factor increases hippocampal GABA and attenuates anxiety behavior via the brain-salivary gland connection in mice^53^. Therefore, it might be possible that the present study contributes not only to find the possible biomarker for sleep disorders but also shines a light on the brain-salivary connection in sleep regulation. Further studies are needed to validate and refine the diagnostic potential of these metabolites.

## Methods

### Animals

Five-week-old male C3H/HeN mice (Japan SLC Inc., Hamamatsu, Japan) were individually housed in plastic cages containing paper-chip bedding and running wheels (SW-15; Melquest Y.K, Toyama, Japan). The mice had free access to a standard diet (AIN-93G: Oriental Yeast Co. Ltd., Tokyo, Japan) and tap water under a 12 h light-12 h dark cycle (lights on at 08:00) for four weeks until daily wheel-running activity reached a plateau. The CSD mice were then exposed to psychophysiological stress for one week to induce CSD^23^. Briefly, paper-chip bedding was replaced with water to a depth of 1.5 cm, which caused the mice to remain on the wheels throughout every day of the study. Wheel-running activity was continuously recorded at 1-min intervals using Chronobiology Kits (Stanford Software Systems, Stanford, CA, USA) and activity data are displayed as actograms. All protocols complied with the guidelines for animal experiments published by the National Institute of Advanced Industrial Science and Technology (AIST) and the ARRIVE (Animal Research: Reporting of In Vivo Experiments) guidelines. The AIST Animal Care and Use Committee approved all experimental protocols (Permission No: #2021-338).

### Sleep recording and analysis

Two EEG electrodes were implanted into the skulls of the mice 1.0 mm anterior to the bregma and 1.0 mm lateral (right) to the midline, and 3.0 mm posterior to the bregma and 1.0 mm lateral (left) to the midline, then fixed with dental cement under anesthesia with 2–4% isoflurane. Two stainless steel wires were then implanted into the neck muscles to collect electromyographic (EMG) signals. Sleep was then recorded by TL11M2-F20-EET telemetric devices (Data Sciences International, St. Paul, MN, USA) that were subcutaneously implanted into the backs of the mice as described^22^. Polygraphic EEG and EMG findings were continuously recorded for the last 72 h of the experiment. Cortical EEG and EMG signals were digitized at a sampling rate of 500 Hz and recorded using Dataquest A.R.T.™ (Data Sciences International). Polygraphic recordings were automatically scored offline in 10-s epochs divided into stages of wakefulness, REM, and NREM sleep using SLEEPSIGN (Kissei Comtec, Nagano, Japan) according to the standard criteria^22^. The defined sleep–wake stages were visually examined and corrected if necessary. Power spectrum density was calculated at ~ 0.48 Hz intervals, then the EEG delta and theta frequency bands were set at 0.5‒4.9 and 5.4‒7.8 Hz, respectively.

### Saliva collection

After one week of CSD, the mice were fasted from 18:00, then saliva secretion was induced by an intraperitoneal injection of pilocarpine hydrochloride (1.5 mg/kg body weight; Fujifilm Wako Pure Chemical Co., Osaka, Japan) at 22:00 (2 h after lights off). Saliva collected from the oral cavity using micropipette tips was immediately stored at −80 °C.

### CE-FTMS analysis

Saliva (40 µL) in Milli-Q water (10 µL) containing internal standards (H3304-1002, Human Metabolome Technologies, Inc. (HMT), Tsuruoka, Yamagata, Japan) was centrifugally filtered through a Ultrafree^®^-MC PLHCC, HMT 5-kDa cutoff filter (MilliporeSigma, Burlington. MA, USA) at 9100×*g*, at 4 °C for 60 min to remove macromolecules.

Metabolomes in the filtrate were analyzed using the *ω Scan* package (Human Metabolome Technologies [HMT] Tsuruoka, Japan) with CE-FTMS as described^54^. Briefly, saliva samples were analyzed by CE-FTMS using an 7100 CE capillary electrophoresis system (Agilent Technologies Inc., Santa Clara, CA, USA) equipped with Q Exactive Plus (Thermo Fisher Scientific Inc., Waltham, MA, USA), a 1260 isocratic HPLC pump, G1603A CE-MS adapter kit, and G1607A CE-ESI–MS sprayer kit (all from Agilent Technologies Inc.). The systems were controlled by MassHunter workstation software for data acquired using a 6200 series TOF and a 6500 series Q-TOF LC/MS version B.08.00 (both from Agilent Technologies Inc.) and Xcalibur (Thermo Fisher Scientific Inc.), and connected by a fused silica capillary (50 μm *i.d. * × 80 cm total length). H3301-1001 and I3302-1023 electrophoresis buffers functioned as electrolytes for cation and anion analyses, respectively (HMT). The spectrometer scanned samples from *m/z* 50‒1000^54^. Peaks were extracted using MasterHands automatic integration software (Keio University, Tsuruoka, Yamagata, Japan) to acquire information about *m/z*, peak area, and migration time (MT)^14^. Signal peaks corresponding to isotopomers, adduct ions, and other product ions of known metabolites were excluded, and the remaining peaks were annotated according to the HMT metabolite database based on their *m*/*z* values and migration times (MTs). Areas of the annotated peaks were then normalized to internal standards and sample volumes to determine relative levels of individual metabolites. Principal component analysis (PCA) of samples proceeded using SampleStat (HMT).

### LC-TOF MS analysis

Saliva (80 µL) was mixed with methanol (240 µL) containing H3304-1002 internal standards (HMT) and centrifuged for 5 min at 2300×*g* and 4 °C. The supernatant was evaporated to dryness under nitrogen and reconstituted in 160 µL of 50% isopropanol (v/v) for metabolome analysis using LC-TOFMS with the HMT *LC* package as described^55, 56^. Briefly, the LC-TOFMS system comprised a 1200 HPLC pump with a 6210 TOF–MS mass spectrometer (Agilent Technologies Inc.). The systems were controlled by MassHunter (Agilent Technologies Inc.) and connected by an ODS column (2 mm *i.d.*  × 50 mm, 2 μm). The spectrometer scanned from *m/z* 50‒1000 and peaks were extracted using MasterHands automatic integration software (Keio University) to generate information about *m/z* values, peak area, and retention time (RT)^14^. Signal peaks corresponding to isotopomers, adduct ions, and other product ions of known metabolites were excluded, and the remaining peaks were annotated according to the *m*/*z* values and RTs in the HMT metabolite database. The areas of the annotated peaks were then normalized to internal standards and sample values to determine relative levels of individual metabolites and PCA were determined using SampleStat.

### Measurement of brain lactate concentrations

Whole brains were dissected at 22:00 and rapidly frozen in liquid nitrogen. Thereafter, frozen tissues (500 mg) were homogenized in ice-cold distilled water (5 mL) and centrifuged for 10 min at 18,000×*g*. Brain concentrations of lactate were then determined in supernatants (20 μL) as described^44^ using a GM7 MicroStat multi-assay analyzer (Analox Instruments, London, UK) calibrated with 8.0 M lactate standard (GMRD-103; Analox Instruments).

### Measurement of blood lactate and IL-6 concentrations

Blood collected in EDTA-coated tubes was immediately separated by centrifugation for 15 min at 5800×*g,* then plasma was stored at −80 °C. Plasma concentrations of lactate and IL-6 were respectively measured using Lactate Assay Kits (BioVision, Inc., Milpitas, CA, USA) and Mouse IL-6 Quantikine ELISA Kits (R&D Systems, Inc., Minneapolis, MN, USA).

### Measurement of blood and brain glycine and sarcosine concentrations

The concentrations of glycine and sarcosine in the plasma and brain were measured by HPLC (NDTS Inc., Hokkaido, Japan) after derivatization using EZ-faast^®^ Amino Acid analysis kits (Phenomenex^®^, Shimadzu GLC Inc., Tokyo, Japan)^57^.

### Data analysis and statistics

We confirmed associations between CSD stress and metabolic pathways in mouse saliva by MESA using MetaboAnalyst 5.0^58^. The metabolites for MESA were selected based on previous findings^59^. Factor loading in PC1 was analyzed using statistical hypothesis tests, and metabolites were considered statistically significant at *q* < 0.05 based on the methods of Benjamini and Hochberg^60^. Metabolites that were significantly positive and negative for PC1 were independently assessed by MESA.

## Supplementary Information

Supplementary Table 1.

Supplementary Table 2.

Supplementary Table 3.

Supplementary Table 4.

Supplementary Figure 1.

## Data Availability

The data presented herein are included in the article and Supplementary Materials.
