# Supplementary material for: Metabolic profiles of saliva in male mouse models of chronic sleep disorders induced by psychophysiological stress
Source: Sci Rep. 2023 Jul 10;13:11156. doi: 10.1038/s41598-023-38289-1 (PMC10333369; doi:10.1038/s41598-023-38289-1)
Supplement: Supplementary file 5 — Supplementary Figure 1. [file 41598_2023_38289_MOESM5_ESM.pdf]

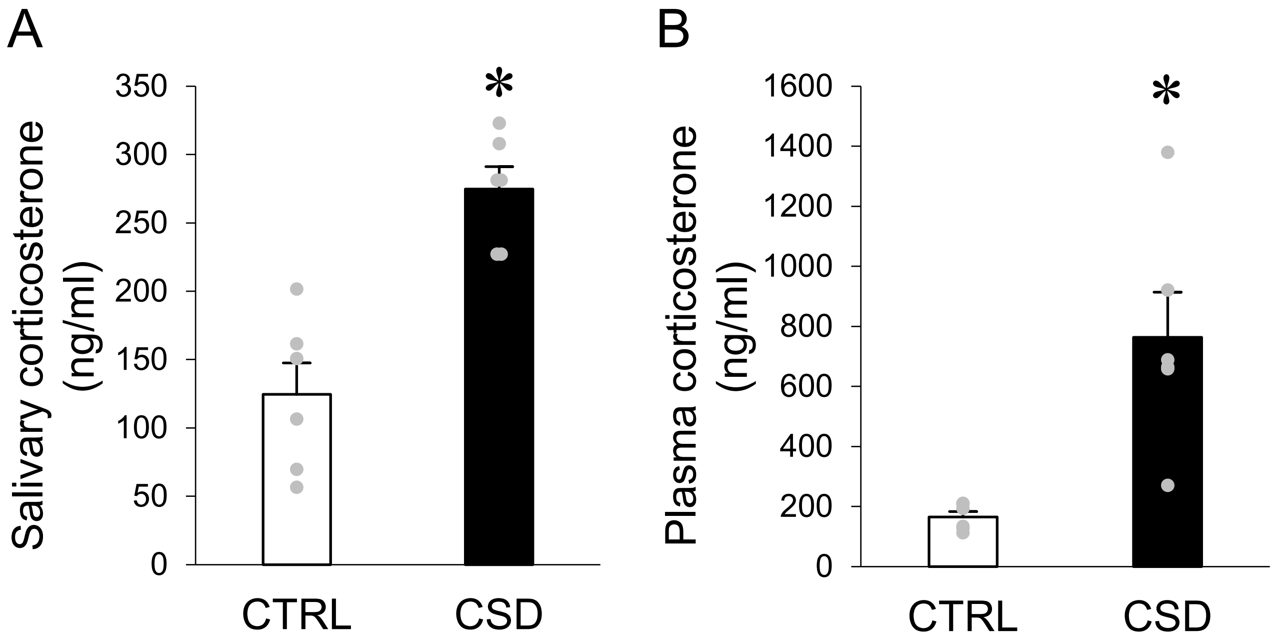

Supplemental Figure 1. Corticosterone concentrations in saliva (A) and plasma (B). Data are shown as means ± SEM (n = 6). Control and CSD mice significantly differed. *$P < 0.01$.
